# The prevalence of hereditary hemorrhagic telangiectasia in Juvenile Polyposis syndrome patients with *SMAD4* mutations

**DOI:** 10.1186/1897-4287-9-S1-O5

**Published:** 2011-03-10

**Authors:** Margaret O’Malley, Lisa LaGuardia, Matthew Kalady, Joe Parambil, Brandie Leach, Charis Eng, James Church, Carol Burke

**Affiliations:** 1 The Sanford R. Weiss, M.D, Center for Hereditary Colorectal Neoplasia, Digestive Diseases Institute, Desk A30, 9500 Euclid Ave., Cleveland Clinic, Cleveland, OH 44195, USA; 2 Respiratory Institute, Cleveland Clinic, 9500 Euclid Ave., Cleveland, OH 44195, USA; 3 Genomic Medicine Institute, 9500 Euclid Ave., Cleveland Clinic, Cleveland, OH, 44195, USA

## Background

Juvenile Polyposis Syndrome (JPS) is defined by the presence of ≥ 5 colorectal juvenile polyps or any number of juvenile polyps in an individual with a family history of JPS. Genetic alterations including either point mutations or large rearrangements in *BMPR1A* or *SMAD4* are found in 50% of affected individuals. Hereditary Hemorrhagic Telangiectasia (HHT) is an autosomal dominant disease diagnosed upon the presence of epistaxis, visceral arteriovenous malformations (AVM) or mucutaneous telangiectasias. HHT is diagnosed when there are ≥ 3 manifestations and is suspected when there are at least 2 manifestations. Most HHT cases are caused by a germline mutation in *ALK1* or *ENG*, members of the TGFβ signaling pathway. Approximately 22% of patients with Juvenile Polyposis Syndrome (JPS) due to a *SMAD4* mutation have been reported to also have HHT [1]. Most prior publications have few patients and no systematic approach to screening, so the true incidence of the combined JPS/HHT syndrome is not known. Our aim was to determine the prevalence of HHT in our patients with JPS with a *SMAD4* mutation including those who underwent systematic screening for AVM’s.

## Methods

JPS patients were identified from a comprehensive polyposis database using Cologene© software. Families carrying a germline *SMAD4* mutation were studied by screening affected patients for cutaneous telangiectases and with cardiac bubble ECHO, CAT scan chest, or MRI of brain for other AVMs.

## Results

Fourteen of 38 JPS families underwent genetic testing. Nine families were identified to have a *SMAD4* mutation. These families include 21 affected relatives, 11 men and 10 women, with a current mean age of 36.3 years (range 4 - 70). Fourteen affected relatives, from 6 families, underwent HHT screening (7 men and 7 women, with a mean age of 35.4 years (range 15 -70). Eleven of 14 (79%) had ≥ 3 HHT manifestations and two of 14 (14 %) had at least 2. In addition, 3 of 7 unscreened affected relatives have presented with at least 2 manifestations of HHT. Of the 24 families that have not had genetic testing or HHT screening one affected family member presented with ≥ 3 HHT manifestations, and two had at least 2 manifestations.

## Conclusion

Greater than 90% of our patients with JPS due to *SMAD4* mutations had clinically diagnosed or suspected HHT. Genetic testing should be performed in all JPS patients. In addition, systematic HHT screening is recommended for JPS patients with *SMAD4* mutations.
